# Glucogallin Attenuates RAW 264.7 Cells from Arsenic Trioxide Induced Toxicity via the NF-ҡB/NLRP3 Pathway

**DOI:** 10.3390/molecules27165263

**Published:** 2022-08-18

**Authors:** Anam Najib Khan, Rajveer Singh, Arka Bhattacharya, Sonu Kumar, Arijit Ghosh, Debasish Nag, Velayutham Ravichandiran, Dipanjan Ghosh

**Affiliations:** 1 Department of Natural Products, National Institute of Pharmaceutical Education and Research, Kolkata 700054, India; 2 Netaji Subhas Chandra Bose Cancer Research Institute, 3081, Nayabad, Kolkata 700094, India

**Keywords:** arsenic trioxide, β-glucogallin, apoptosis, reactive oxygen species, mitochondrial dysfunction, antioxidant

## Abstract

Chronic arsenic (As) poisoning is mostly due to subsoil water contaminated with As and its salts. Exposure to As has been found to cause an elevation in reactive oxygen species (ROS), leading to the damage of DNA and proteins, and it also causes immunotoxicity. Treatment regimens are primarily based on chelation therapy and amino acid and vitamin supplementations. Recent studies have established that natural products display effective and progressive relief from arsenicosis without any side effects. β-glucogallin (BGG), a gallo-tannin natural product, is reported to possess anti-oxidant and anti-inflammatory properties. In the present study, we aim to observe the protective role of BGG against As-induced cytotoxicity, apoptosis, mitochondrial dysfunction, and the underlying mechanisms in RAW 264.7 macrophage cells. We found that BGG alleviates As-induced ROS, apoptosis, and mitochondrial dysfunction in RAW 264.7 macrophage cells. Thus, BGG can be used therapeutically to prevent As-induced toxicity.

## 1. Introduction

Arsenicosis is defined as a chronic health condition arising from prolonged or high-level ingestion of As above a safe dosage [1]. More than 140 million people across the globe are potentially exposed to elevated levels of As, mostly from Asia and Latin America. The common source of As-toxicity is As-contaminated drinking water. The exposure of As through drinking water is increasing daily due to industrial contamination and the overdrawing of underground water for irrigation [2]. Food crops contaminated with As are also responsible for the onset of various diseases, such cancer, skin problems, etc.

Exposure to As has been found to cause an elevation in reactive oxygen species (ROS) such as peroxyl radicals (ROO^•^), superoxide anion radical (O_2_^•–^), singlet oxygen (^1^O_2_), hydroxyl radical (HO^•^), hydrogen peroxide (H_2_O_2_), and dimethyl As radical [(CH_3_)_2_As^•^], leading to the damage of DNA and proteins responsible for altering the cellular architecture, permeability, and cell survival [3]. As has been found to activate a series of downstream signaling cascades and to further disrupt signaling related to cell growth, proliferation, and apoptosis [1]. The presence of urinary As and metabolites, such as MMA(III), or methylarsonic acid, and DMA(III), or dimethylarsinic acid, are considered to be better indicators of As exposure [4]. The current management strategies against As-induced toxicity include chelation therapy, filtration, supplements such as amino acids and vitamins, and micronutrients such as selenium, zinc, calcium, and magnesium. Chelation therapy was found to be the most effective treatment against As-induced toxicity. Chelation therapy generally includes the use of chelating agents such as British anti-lewisite, sodium 2,3-dimercaptopropane-1-sulfonate, meso 2,3-dimercaptosuccinic acid, etc. [5].

The previous study revealed that many medicinal plants have ameliorative potential against As-induced toxicity. The plants, mentioned in Ayurveda (the traditional system of Indian medicine) and worldwide, include *Withania somnifera*, *Mentha piperita*, *Emblica officinalis*, *Azadirachta indica*, *Boerhavia diffusa*, *Camellia sinensis*, *Vitis vinifera*, *Terminalia arjuna*, and *Moringa oleifera*. Moreover, quercetin, resveratrol, rutin, β-carotene, leutin, diallyl trisulfide, silibinin, naringenin, curcumin, and vitamins such as ascorbic acid, α-tocopherol, and all-trans-retinoic acid were also found to possess ameliorative potential against As-induced toxicity [6,7,8,9,10,11,12].

**Figure 1:**
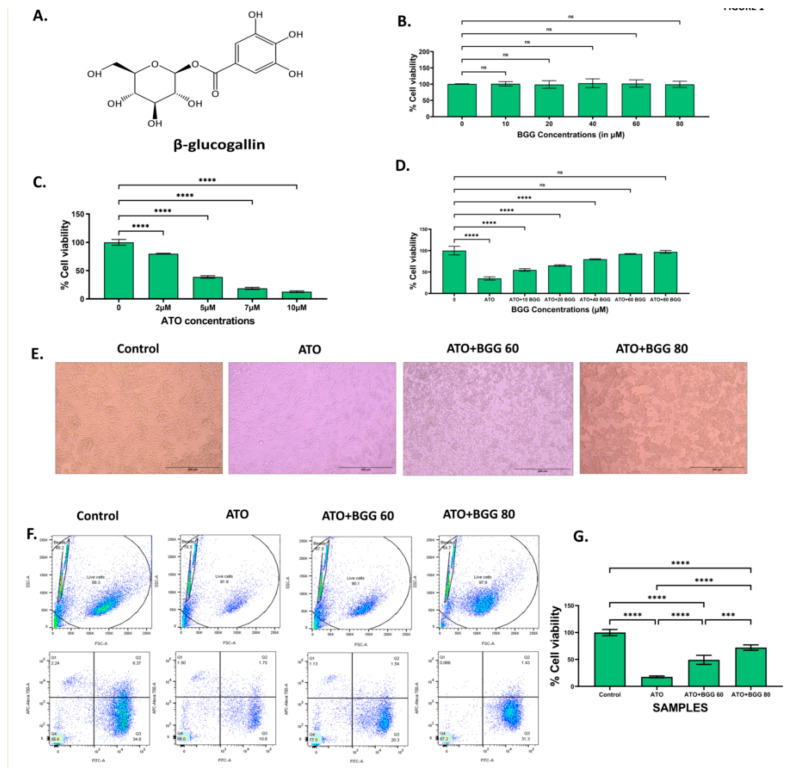
(**A**) β-glucogallin (BGG) chemical structure. (**B**)% Cell viability of different concentrations of BGG on RAW 264.7 for 24 h measured by MTT assay. (**C**) % Cell viability of different concentrations of arsenic trioxide (ATO) on RAW 264.7 for 24 h measured by MTT assay. (**D**) Protection of RAW 264.7 cells by pre-treatment with BGG measured by MTT assay. Cells were pre-treated with different BGG concentrations for 1 h, followed by ATO (5 μM) treatment for 23 h. (**E**) Cell images representing the morphology of RAW 264.7 cells, scale bar 200 µm. (**F**)% Cell viability measured by flow cytometry with Calcein-AM and PI. (**G**) Graph represents the percentage of cell viability, mean ± SD of two experiments. For (**B**–**D**) results are mentioned as mean ± SD (*n* = 3), *p*-value ≤ 0.05 (*** *p* < 0.001, and **** *p* < 0.0001). ns = non-significant.

β-glucogallin (BGG) is a natural compound formed from gallic acid and β-D-glucose (Figure 1A) [13,14]. Natural sources of BGG include amla fruit (*Emblica Officinalis*), North American white oak (*Quercus alba*), and European red oak (*Quercus robur*) [15,16]. Glucogallin acts as both an acyl acceptor and donor throughout the pentagalloylglucose bio-synthetic pathway, revealing its energy-rich activated compound nature [16]. Glucogallin is one of the main plant polyphenolic anti-oxidants linked with various positive effects on human health [14,17]. It is studied majorly as an aldose reductase inhibitor [18]. Human aldose reductase enzyme (AKR1B1) functions in the polyol pathway as an NADPH–dependent enzyme, catalyzing the reduction of glucose to sorbitol and then to fructose by sorbitol dehydrogenase [19]. Due to its ability to scavenge free radicals, glucogallin is believed to provide protection against several diseases, such as diabetes, glaucoma, hepatic damage, and UV induced adversaries [18,20,21,22,23].

The present study aims to establish the anti-oxidant activity of β-glucogallin (BGG) in vitro against As-induced toxicity. In this study, we investigate the activity of BGG against As-induced toxicity in RAW 264.7 macrophage cells. Our results show that BGG attenuated ROS production, mitochondrial dysfunction, apoptosis, and pro-inflammatory cytokine production, and also inhibited the activation of the inflammatory and pyroptotic pathways NF-κB and NLRP3.

## 2. Materials and Methods

### 2.1. Materials

Cell culture reagents Dulbecco’s modified Eagle’s medium (DMEM,11885-084), fetal bovine serum (FBS,10270-106), antibiotic-antimycotic solution (100X) (Cat. 15240062), and phosphate-buffered saline (PBS) (BP39920) were purchased from Gibco-BRL (Life Science Technologies). β-glucogallin (BGG) (Cat. G416000) was purchased from Toronto Research Chemicals (Toronto, Ontario, Canada). Arsenic trioxide, sodium nitrite (7632000), Griess reagent (G4410-10G), DCFDA (D6883), DMSO (D9170-1VL), and the protease inhibitor cocktail (P8340) were purchased from Sigma Aldrich, USA. Propidium iodide (PI) (640914) and annexin V (640914) were purchased from Biolegend.(3-(4,5-dimethylthiazol-2yl-)-2,5-dihenyl tetrazolium bromide (MTT dye)(298-93-1)was purchased from HIMEDIA. Calcein-AM (C3100MP), DAPI (D1306), mitosox red (M36008), and BCA protein assay reagent (23225) were purchased from Thermo Scientific. Rabbit antibodies for p-p65 (Ser536) and Alexa Fluor 488 anti-rabbit (4412S) were purchased from Cell Signaling.. PARP1 (A19596) was purchased from Abclonal. NLRP3 (IMG-6668A) and IL-1 beta (NB600-633) were acquired from Novus Biologicals. For western blot rabbit antibodies, BAX (A19684) was purchased from Abclonal. BCL-XL (2764S) was purchased from Cell Signaling. ECL for western blotting was purchased from Biorad.

### 2.2. Cell Culture

RAW 264.7 macrophage cells were grown in a high-glucose Dulbecco’s modified eagle medium containing 10% heat-inactivated FBS and 1% anti–anti penicillin–streptomycin cocktail. The medium was changed every two days, and cultures were maintained at 37 °C in a humidified CO_2_ incubator with 5% CO_2_**.**

### 2.3. Cell Viability Assay

MTT is a sensitive indicator of cell viability. This assay uses the reducing property of MTT to color insoluble formazan crystals by viable mitochondrial cells [24]. Briefly, the RAW 264.7 cells were seeded in a 96-well plate at a density of 1 × 10^4^ cells/well and incubated for 24 h at 37 °C in 5% CO_2_. Further, the cells were treated with different concentrations of BGG (10, 20, 40, 60, and 80 μM) and ATO (2, 5, 7, and 10 μM) separately for 24 h. The cells were pre-treated with BGG (10, 20, 40, 60, and 80 μM) 1 h prior to ATO (5 μM) treatment for 24 h. After 24 h of treatment, 10 μL (5 mg/mL) of MTT was added, and the cells were incubated at 37 °C in 5% CO_2_ for 4 h. Following that, 200 μL of dimethyl sulfoxide (DMSO) was added to each well to solubilize the formazan crystals, and absorbance was read at 570 nm using a microplate reader (Synergy H1 microplate reader, Biotek).

### 2.4. Calcine-AM and PI staining

Calcein-AM and PI is a reliable method for the detection of living and apoptotic cell populations [25]. Briefly, the cells were seeded in a six-well plate at a density of 3 × 10^5^ cells/well for 24 h of incubation. The cells were pre-treated with BGG (60, 80 μM) 1 h prior to ATO (5 μM) treatment for 24 h. Further cells were subjected to 5 μM Calcein-AM for 30 min, followed by incubation with 250 μg/mL PI. Data were generated using BD LSRFortessa™ flow cytometer and analyzed using FlowJo™ v10.

### 2.5. Quantification of Intracellular ROS Formation

DCFDA, a fluorogenic dye, was used for the measurement of intracellular ROS. The RAW 264.7 cells were seeded in a 12-well plate at a density of 1 × 10^5^ cells/well and incubated for 24 h at 37 °C in 5% CO_2_. The cells were pre-treated with BGG (60, 80 μM) 1 h prior to ATO (5 μM) treatment for 24 h. After 24 h of treatment, cells were incubated with 10 μM of DCFDA at 37 °C for 30 min in dark conditions. Mitochondrial-derived ROS (mtROS) were measured using the MitoSOX^TM^ Red with a concentration of 5 μM. After staining, the cells were washed and acquired by BD LSRFortessa™ flow cytometer and further analysis was done by FlowJo™ v10 software.

### 2.6. Measurement of Mitochondrial Membrane Potential by JC-1

Mitochondrial membrane potential was monitored using a fluorescent probe JC-1. Briefly, the RAW 264.7 cells were seeded in confocal plates for 24 h. After 24 h of treatment, the cells were pre-treated with BGG (60, 80 μM) 1 h prior to ATO (5 μM) treatment for 24 h. Next, the cells were washed twice with 1X PBS and stained with JC-1 solution at a concentration of 2 μM, followed by incubation at 37 °C for 30 min in the dark condition. In healthy cells, JC-1 accumulates at higher concentrations and forms J-aggregates emitting red fluorescence, indicating a higher mitochondrial membrane potential. However, in unhealthy or apoptotic cells, JC-1 at a low concentration appears as J-monomers emitting green fluorescence, indicating the low mitochondrial membrane potential. The red–green intensity ratio decreased, indicating low mitochondrial membrane potential, and images were captured using Leica confocal laser scanning microscopy.

### 2.7. Measurement of Intracellular GSH Activity

The levels of GSH (Arbor assays K006-F5) were measured with commercial assay kits according to manufacturer’s protocol. The fluorescence was measured at 510 nm emission and 410 nm excitation wavelength using a microplate reader (Synergy H1 microplate reader, Biotek).

### 2.8. Cytometric Bead Array (CBA) for Extracellular Cytokines

Extracellular cytokines were measured using a BD CBA mouse Th1/Th2/Th17 cytokine kit. The levels of Th1 (TNF, and IFN-α), Th2 (IL-4, IL-6, and IL-10), and Th17 (IL-17 A) cytokines were measured according to the manufacturer’s protocol. Further, 50 µL of capture beads were mixed with 50 µL of supernatant of treated RAW macrophages. The samples were loaded into the assay tubes; the tubes were incubated in the dark for 150 min. Next, the samples were washed with 300 µL of wash buffer and were analyzed using BD LSRFortessa™ flow cytometer.

### 2.9. Intracellular IL6 and IFN Production

The measurement of the levels of intracellular IL-6 and IL-10 were performed according to manufacturer’s protocol [26]. The samples were analyzed using BD LSRFortessa™ flow cytometer, and data were analyzed using FlowJo™ v10 software.

### 2.10. Apoptotic Cell Quantification by Annexin V and PI

The quantification of apoptotic cells was performed using annexin V-FITC and propidium iodide (PI) double staining. The RAW 264.7 cells were seeded in a 12-well plate at a density of 1 × 10^5^ cells/well for 24 h. The cells were pre-treated with BGG (60, 80 μM) 1 h prior to ATO (5 μM) treatment for 24 h. The cells were washed with 1XPBS twice, detached with PBS-EDTA, and centrifuged at 500× g for 5 min at 4 °C. Furthermore, the cells were then stained with 2 μL of annexin V and 4 μL of PI. The cells were analyzed by flow cytometry (BD LSRFortessa) for quantification of apoptosis, and 20,000 events were counted and analyzed for each sample.

### 2.11. Western Blot Analysis

The total protein from the RAW 264.7 cell was isolated using the Total Protein Extraction Kit, followed by protein concentration quantification by the BCA assay. The separation of proteins was performed by electrophoretic separation in 10% SDS-PAGE gel, and then transferred to the PVDF membrane. Blocking was carried out at room temperature using skimmed milk in PBST for 1 hour, and then probed at 4 °C overnight with primary antibodies while shaking. The membranes were then probed with secondary antibodies, and enhanced chemiluminescence (Millipore) was used to assess the protein levels using the Chemi-Doc Imaging System (Bio-Rad, Hercules, CA, USA).

### 2.12. Study of PARP1/NFҡβ/NLRP3//IL-1β Expression by Confocal Microscopy

For the immunofluorescence staining assay, the RAW 264.7 cells were seeded in confocal plates at a density of 1 × 10^4^ cells/plate for 24 h. The cells were pre-treated with BGG (60, 80 μM) 1 h prior to ATO (5 μM) treatment for 24 h. Next, the cells were washed with 1X PBS twice and were fixed with 4% formaldehyde in 1X PBS for 15 min. The fixed cells were permeabilized with 0.5% Triton-X100 in PBS for 15 min and blocked with 3% BSA for 1 h. Furthermore, the cells were then incubated with the appropriate primary antibodies (PARP1, Pp-65, NLRP3, IL-1β) overnight at 4 °C. The cells were washed three times with 1X PBS, stained with fluorescent secondary antibodies, and incubated for 1 h. Next, the cells were washed with 1X PBS, and the cell nucleus was stained with DAPI. Images were captured by Leica confocal laser scanning microscopy.

### 2.13. Statistical Analysis

The statistical significance was evaluated with data from at least three independent experiments. Statistical analysis was carried out in R; for the pairwise-statistical test, one-way Anova was used; *p* ≤ 0.05 is considered significant (* *p* ≤ 0.05, ** *p* < 0.01, *** *p* < 0.001, and **** *p* < 0.0001,ns=non-significant), and graphs were plotted using ggplot2. MFIs (mean fluorescence intensity) were taken from five different fields.

## 3. Results

### 3.1. Effect of BGG against ATO Induced Cell Death

To check the anti-oxidant effect of BGG against ATO induced toxicity on RAW 264.7 macrophages cells, the cells were treated with different concentrations of BGG (10, 20, 40, 60, and 80 μM) and ATO (5 μM) separately. The cells were pre-treated with BGG (10, 20, 40, 60, and 80 μM) 1 h prior to ATO (5 μM) treatment for 24 h. After 24 h of treatment, no significant change in % cell viability was observed in the BGG-treated cells, as compared with that of the control (Figure 1B). However, ATO (5 μM) showed a significant decrease in % cell viability as compared with that of the control group (Figure 1C). Pre-treatment with BGG (10, 20, 40, 60, and 80 μM) 1 h prior to ATO (5 μM) treatment shows a significant increase of the % cell viability in a dose-dependent manner, as compared with that of the ATO treated group alone in RAW 264.7 macrophage cells (Figure 1D). To analyze the protective effect of BGG on the ATO-degraded morphology of RAW 264.7, the cells were pre-treated with BGG for 1 h, followed by ATO for 23 h. After 23 h of ATO exposure, changes in morphology were clearly visible. The cells were detached from the surface of the plate, and had lost their shape. BGG pre-treatment reverses the effect of ATO; those cells were attached to the surface of the plate, and remained in their original shape (Figure 1E).

Next, we checked the % live cells with Calcein-AM and PI. Calcein-AM is a cell-permeant dye that can be used to determine the % of live cells. ATO (5 μM) alone decreases the % of live cells, thus reducing the Calcein-AM fluorescence and increasing the PI fluorescence, compared with the control. Pre-treatment with BGG (60, 80 μM) resulted in increase of the Calcein-AM fluorescence and decrease of the PI fluorescence in a dose-dependent manner, indicating the increasing % of live cells as compared to the control in the RAW 264.7 macrophage cells (Figure 1F,G).

### 3.2. Effect of BGG against ATO-Induced Intracellular ROS Formation

**Figure 2:**
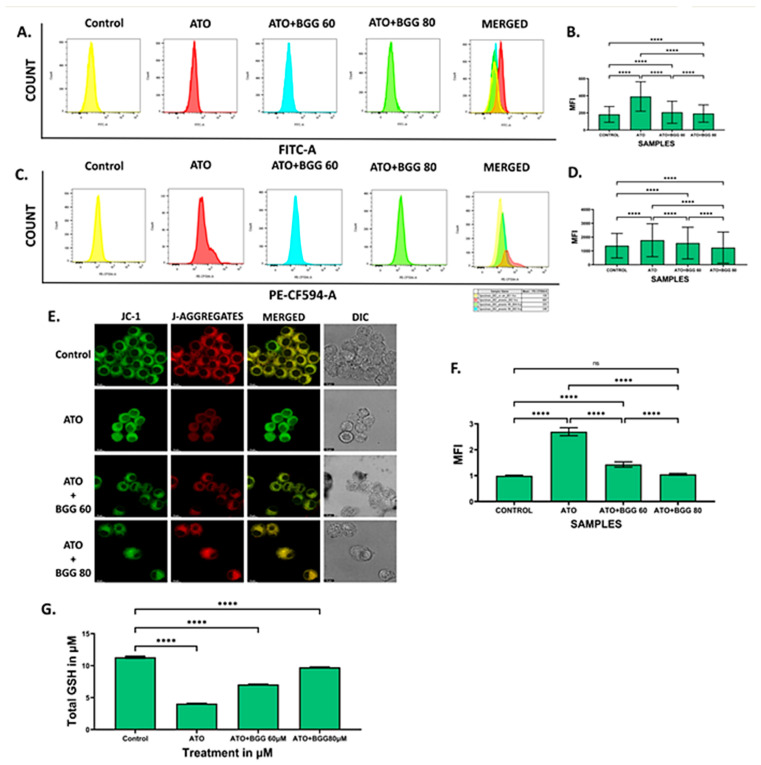
BGG attenuates the ATO-induced ROS, mitochondria superoxide, and mitochondria membrane potential depolarization. RAW 264.7 cells were pre-treated with 60 μM and 80 μM of BGG for 1 h, followed by ATO (5 μM) treatment for 23 h of stimulation; (**A**) ROS measurements by FACS after staining with DCFDA, (**B**) graph represents the mean fluorescence intensity (MFI) ± SD of the DCFDA experiments, (**C**) FACS assay for the measurement of superoxide production after staining with MitoSox, (**D**) graph represents the mean fluorescence intensity (MFI) ± SD of MitoSox experiments, (**E**) JC-1 staining, confirming that BGG attenuates the ATO degraded membrane potential measured by confocal microscopy in RAW 264.7 cells, (**F**) graph represents the mean fluorescence intensity of showing a ratio of JC-1 monomer vs. JC-1 aggregates, and (**G**) GSH assay. (Mean ± SEM) (*n* = 3), *p*-value (**** *p* < 0.0001, ns = non-significant).

A DCFDA probe measures the intracellular ROS generation induced by 5 μM of ATO for 24 h and estimated by flow cytometry. After 24 h of treatment, ATO significantly induces intracellular ROS in RAW 264.7 macrophage cells, compared with the control. However, BGG (60 μM, 80 μM) treatment significantly decreased the intracellular ROS generation in a dose-dependent manner induced by ATO. This result indicates that BGG can scavenge intracellular ROS generation induced by ATO (Figure 2A,B). We also investigated the superoxide production in the mitochondria of live cells by MitoSOX^TM^ Red. BGG (60 μM, 80 μM) treatment significantly decreased the intracellular ROS generation in a dose-dependent manner induced by ATO (Figure 2C,D).

### 3.3. Effect of BGG on ATO-Induced Mitochondrial Dysfunction

The mitochondrial membrane potential was measured by JC-1. In ATO (5 μM)-treated RAW 264.7 macrophages, a decrease in the red–green intensity ratio and increase in (J-monomers) green fluorescence was observed compared with the control cells, indicating the low mitochondrial membrane potential. However, BGG (60, 80 μM) treatment results in a significant increase in the red–green intensity ratio and more (J-aggregates) red fluorescence was observed as compared with ATO treated cells alone, indicating the improvement in mitochondrial membrane potential in a dose-dependent manner. The results suggest that BGG improves the ATO-induced mitochondrial membrane depolarization in RAW 264.7 macrophage cells (Figure 2E,F).

### 3.4. Effect of BGG on GSH Activity

We quantified GSH using a kit from Arbor following the manufacturer’s protocol. We observed that ATO (5 μM) decreased the total GSH compared with the control in cell lysate. However, after treatment with BGG (60, 80 μM), a significant increase in total GSH was observed in a dose-dependent manner in cell lysate, indicating that BGG increases the activity of anti-oxidant enzyme GSH (Figure 2G)

### 3.5. Effect of BGG against ATO-Induced Pro-Inflammatory Cytokines Productions and Inflammation in RAW 264.7 Macrophages Cells

#### 3.5.1. Extracellular Screening of Interleukins

**Figure 3:**
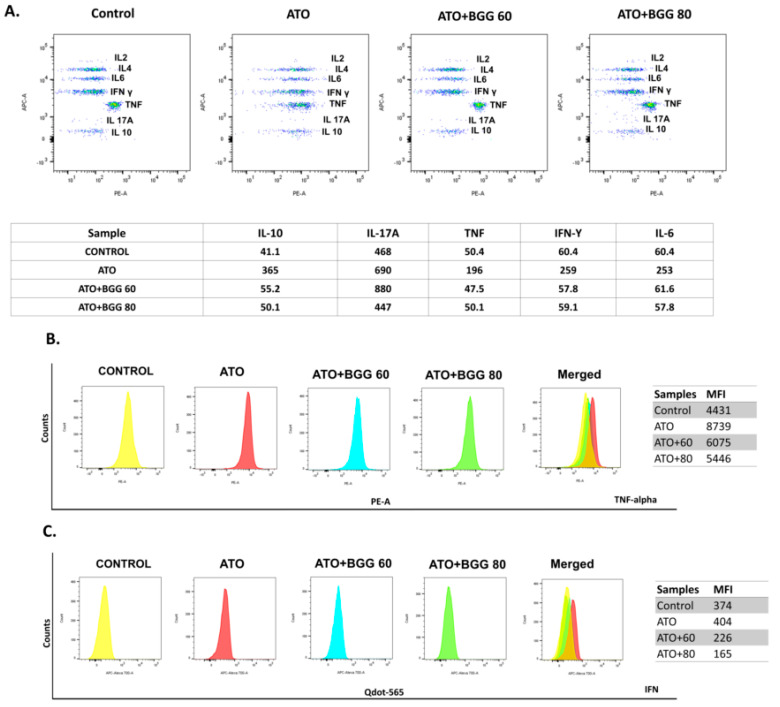
Extracellular cytokine interleukins measured by CBA bead assay, using flow cytometry in (**A)** RAW 264.7. Intercellular cytokines measurements by flow cytometry (**B**) TNF-alpha and (**C**) IFN-gamma.

The change in the interleukin excretion by the RAW 264.7 cells macrophages in vitro was examined using a cytometric bead array (CBA). CBA is a useful technique that allows for the simultaneous detection of various analytes in small volumes with more accuracy as compared with that of conventional immunoassays. After 24 h of treatment, the RAW 264.7 macrophages treated with ATO (5 μM) alone showed a significant increase in interleukin levels of IL-6, IFN-γ, TNFs, IL-17A, and IL-10, as compared with that of the control. However, BGG (60, 80 μM) treatment was shown to cause a significant decrease in the interleukin levels of IL-6, IFN-γ, TNFs, IL-17, and IL-10 in a dose-dependent manner, indicating that BGG attenuates ATO-induced extracellular pro-inflammatory cytokine production (Figure 3A).

#### 3.5.2. Intracellular Staining of Interleukins

To evaluate the immunomodulatory effect of BGG against As-induced toxicity in RAW 264.7 cells, we performed an intracellular flow cytometric examination of TNFs and IFN expression in RAW 264.7 macrophage cells. After 24 h of treatment, in the ATO (5 μM)-treated RAW 264.7 macrophages, a significant increase in interleukin expression of the TNFs and IFN was observed, as compared with that of the control. However, after BGG (60, 80 μM) treatment, a substantial decrease in the interleukin expression of TNFs and IFN was observed in a dose-dependent manner, indicating that BGG attenuates ATO-induced intracellular pro-inflammatory cytokine production (Figure 3B,C).

### 3.6. BGG Prevents ATO-Induced Apoptosis

**Figure 4:**
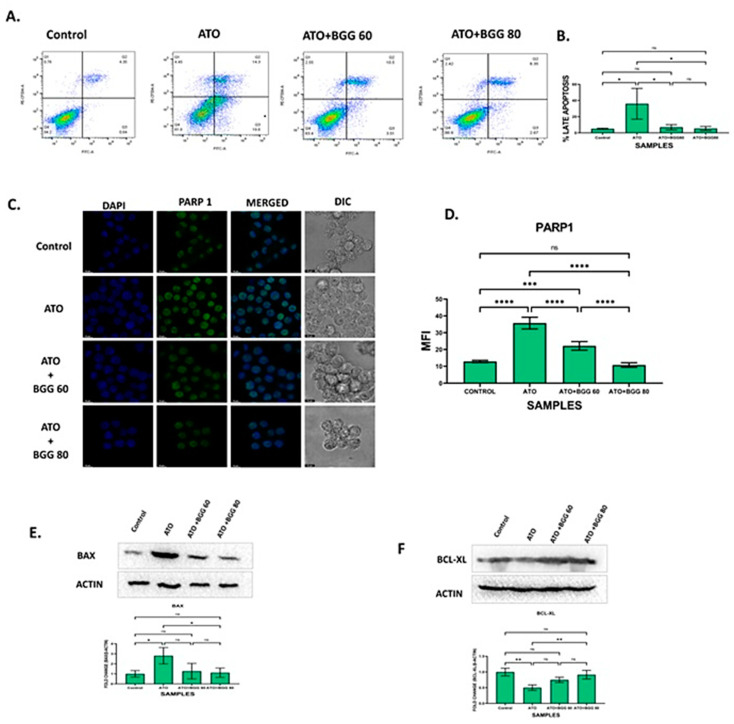
BGG attenuates the ATO-induced apoptosis in RAW 264.7. (**A**) BGG reversed the ATO-induced early and late apoptosis in the RAW 264.7 cells stained with Annexin V/PI, analyzed by flow cytometry. (**B**) Graph represents the percentage of late apoptosis, mean ± SD of two independent experiments. (**C**) Immunofluorescence staining represents the BGG attenuates the ATO-induced PARP1 expression, measured by confocal microscopy scale bars: 10µm. Western blot analysis of (**D**) graph represents the mean fluorescence intensity of PARP1 (**E**) BAX (**F**) BCL-XL. Results are mentioned as mean ± SD (* *p* ≤ 0.05, ** *p* < 0.01, *** *p* < 0.001, and **** *p* < 0.0001, ns = non-significant) (*n* = 3 independent experiments).

The percentage of apoptotic cells was quantified by annexin V and PI dual staining. After 24 h of treatment, ATO (5 μM) alone induced late apoptosis from 4.35% to 14.3% and necrosis from 0.76 to 4.45% in RAW 264.7 macrophage cells. In contrast, BGG treatment decreased late apoptosis up to 10.5% with BGG 60 μM and 8.35% with BGG 80 μM and necrosis up to 2.55% with BGG 60 μM and 2.42% with BGG 80 μM induced by ATO in the cells. The results indicate that BGG attenuates ATO-induced apoptosis in a dose-dependent manner (Figure 4A,B).

### 3.7. Effect of BGG on ATO and Protein Expression of BAX and BCL-XL

ATO treatment significantly increased the protein expression of the pro-apoptotic marker BAX and decreased the expression of the anti-apoptotic marker BCL-XL. However, BGG (60, 80 μM) treatment restored the protein levels of these markers of BAX and BCL-XL (Figure 4E,F).

### 3.8. Effect of BGG on ATO-Induced p-65/NLRP3/PARP1/IL-1β Pathway Activation in RAW 264.7 Macrophages

**Figure 5:**
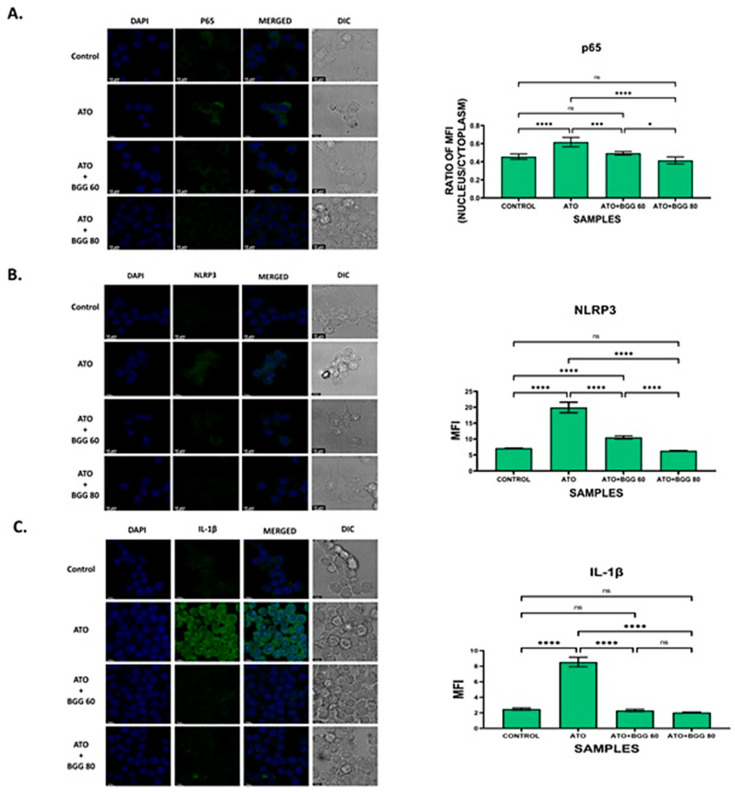
Immunofluorescence staining images demonstrate that BGG reversed the ATO-induced inflammation in the RAW 264.7 cells, measured by confocal microscopy. (**A**) Representation of the translocation of p-65 into the nucleus in RAW 264.7. The histogram represents the ratio of mean fluorescent between nucleus and cytoplasm. (**B**) Immunofluorescence staining shows that BGG protects the ATO-induced inflammasome formation in RAW 264.7 analyzed by NLRP3 (**B**) and IL-1 beta (**C**). Results are mentioned as mean ± SD, (* *p* ≤ 0.05, *** *p* < 0.001, and **** *p* < 0.0001, ns = non-significant) (*n* = 3 fields). Scale bars: 10 µM.

To evaluate the anti-apoptotic activity of BGG against ATO-induced toxicity, we performed immunofluorescence staining of apoptotic markers such as PARP1, P-65, NLRP3, and IL-1β. The immunofluorescence staining assay of PARP1 indicated that ATO (5 μM) increased the accumulation and condensation of PARP1 in the RAW 264.7 macrophages. After treatment with BGG (60, 80 μM), a decrease in the accumulation of PARP1 was observed in the RAW 264.7 macrophages (Figure 4C,D). IKKα, an inhibitor of IκBα, is activated by extracellular signals, which further leads to IKKα activation and the phosphorylation of IκBα, which can then be polyubiquitinated and degraded, leading to the translocation of p-65 from the cytoplasm to the nucleus and thus resulting in the release of pro-inflammatory cytokines such as IL-1β, IL-6, and TNF-α, which cause further inflammation [27,28]. Furthermore, we examined the expression of p-65 with immunofluorescence staining and found that p-65 translocated from the cytoplasm to the nucleus in cells treated with ATO (5 μM) alone, indicating the increased p-65 expression in the nucleus. However, after treatment with BGG (60, 80 μM), a decrease in p-65 expression in the nucleus and an increase in the cytoplasm was observed which was dose-dependent, indicating that BGG attenuates ATO-induced p-65 translocation in the nucleus (Figure 5A).

In order to investigate the role of the NLRP3 inflammasome pathway (the main pathway involved in pyroptosis) [29], an immunofluorescence assay for NLRP3 was performed. The results show that the RAW 264.7 macrophages treated with ATO (5 μΜ) alone lead to NLRP3 translocation from the nucleus to the cytoplasm, which further results in pyroptosis. However, after treatment with BGG (60, 80 μΜ), a significant decrease in NLRP3 expression in the cytoplasm and an increase in the nucleus were observed which were dose-dependent, indicating that BGG attenuates ATO-induced NLRP3 translocation in the cytoplasm. The results suggest that BGG attenuates ATO-induced oxidative stress through the NLRP3 signaling pathway (Figure 5B).

We further investigated the expression of IL-1β, and found that ATO (5 μM) increased the expression of IL-1β. In contrast, treatment with BGG (60, 80 μM) decreased IL-1β expression in a dose-dependent manner, and thus protected against IL-1β-induced inflammation (Figure 5C).

## 4. Discussion

Arsenic is present in ground water in high concentrations in many third world countries, such as India, Bangladesh, Chile, Argentina, Mexico, etc. Human exposure to As comes from contaminated water used for drinking, food preparation, and irrigation [30]. Increasing evidence indicates that As affects the immune system, but specific effects on the immune system are not fully understood [31]. Chronic exposure to As impairs vital immune responses and can increase the risk factors for various chronic diseases, such as cancer and NASH [32]. As disrupts the function of macrophages, the key players of innate immunity, and thus it causes immunosuppression and immunotoxicity [33]. Low micromolar concentrations of As are reported to inhibit in vitro macrophagic differentiations of human blood-derived monocytes [34]. Since no drug or therapy has been established other than chelation therapy for As preventive contamination strategies, there is an urgent need for a drug with less toxicity that can be used against As toxicity [35]. Many natural products, such as rutin, quercetin, curcumin, etc. show promising results against As-induced toxicity [12]. In this work, we used BGG, an anti-oxidant, to protect against ATO-induced toxicity towards RAW 264.7 macrophage cells.

The cytotoxic effects of As are well documented [36]. In our study, we have also observed As cytotoxicity in RAW 264.7 cells when they were treated with ATO and pre-treatment with BGG can protect RAW 264.7 cells from As-induced cell death in a concentration-dependent manner. Although the toxic mechanism of As is not fully understood, it is believed that oxidative stress mediated by reactive oxygen species (ROS) is a common attribute of As toxicity. Exposure induces the generation of intracellular reactive oxygen species (ROS), which mediate changes to cell behavior by altering signaling pathways and epigenetic modifications or causing direct oxidative damage to molecules [37]. Nicotinamide adenine dinucleotide phosphate (NADPH) oxidase (Nox), a membrane-associated enzyme, is involved in ROS generation in response to arsenic [38]. ROS is essential in metabolic processes related to photoprotection, tolerance to various types of stress, and the elimination of pathogens [39]. Disproportion between ROS generation and its elimination gives rise to oxidative stress. Anti-oxidants that potentially reduce ROS levels have been shown to alleviate As-induced lesions [40]. In this study, we found that pre-treatment with BGG can attenuate the excess ROS production by ATO in macrophage cells and thus protect them from its toxicity.

Mitochondria play a pivotal role in energy metabolism, and are the primary site of ROS generation through the electron transport chain. Arsenic has been reported to cause some toxic effects by targeting mitochondria [41]. Arsenic causes mitochondrial oxidative stress and dysfunction by various mechanisms, such as altered membrane potentials, increased oxidative stress, impaired energy metabolism, and deregulated Ca^2+^ homeostasis [42]. Evidence supports the role of mitochondrial dysfunction and increased oxidative stress in As-induced neurotoxicity. Our data shows that ATO increases the superoxide production by mitochondria, which was measured by MitoSox staining. BGG decreased the elevated superoxide production. We also studied mitochondrial membrane dysfunction, where JC-1 accumulates in healthy cells at higher concentrations and forms J-aggregates emitting red fluorescence, indicating a higher mitochondrial membrane potential. In unhealthy or apoptotic cells, JC-1 at low concentration appears as J-monomers and emits green fluorescence, indicating the low mitochondrial membrane potential. Our results suggest that BGG significantly reverses the loss of mitochondrial transmembrane potential caused by arsenic.

GSH (an anti-oxidant molecule present in the cell) and other anti-oxidant enzymes are involved in the process of protection from oxidative damage. Arsenic toxicity has been reported to decrease the cellular levels of GSH and other anti-oxidant enzymes [43]. We foundthat ATO-treated RAW 264.7 cells significantly decreased glutathione. Our result suggests that BGG can increase the GSH, which could be the probable mechanism of protection activity of BGG.

Oxidative stress can lead to apoptosis in the cell. From previous studies it is well known that As-induced apoptosis is dependent on the mitochondrial intrinsic pathway and ROS generation [41]. Here, we investigated ATO-induced apoptosis by flowcytometry analysis after staining the cells with annexin V-FITC and PI, and found that BGG attenuated ATO-induced late apoptosis in RAW 264.7 macrophage cells. Western blot analysis showed that ATO increased pro-apoptotic markers such as BAX and decreased anti-apoptotic BCL-XL, whereas BGG treatment restored these markers’ expression. Confocal imaging of immuno-stained cells showed that BGG lowered the As-induced PARP1 concentration. As also causes cytotoxicity by regulating the functions of the nuclear factor-кB (NF-кB), which regulates normal cell growth through cell development, proliferation, differentiation, and apoptotic cell death [44]. One mechanism underlying this inflammatory activity of NF-кB is the nuclear translocation of p-65, which is reported to cause the release of pro-inflammatory cytokines and cell death. Here we investigated the translocation of P-65, and found that BGG attenuates As-induced pP-65 translocation in the nucleus and thus inhibits the inflammatory response and apoptosis (Figure 5A).

**Figure 6:**
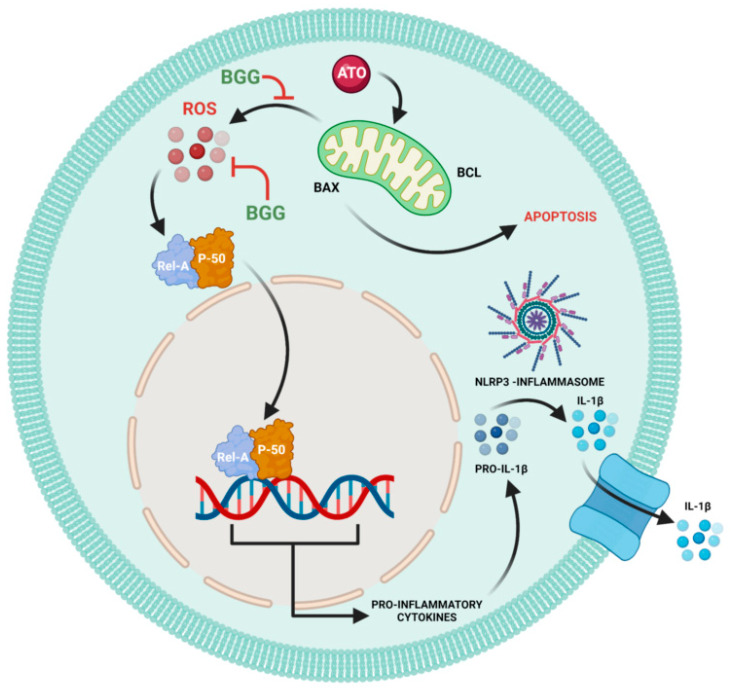
Schematic pathway of BGG attenuates ATO-induced toxicity in RAW 264.7 macrophages.

Mitochondrial reactive oxygen species (mtROS) produced from dysfunctional mitochondria have been linked to NLRP3 activation through multiple mechanisms and the release of pro-inflammatory cytokines [45]. Arsenic exposure has been shown to cause inflammation [46]. Here, we investigated the accumulation of NLRP3 and found that BGG attenuates ATO-induced accumulation in cells. Furthermore, we also investigated the expression of pro-inflammatory cytokine IL-1β, and found that BGG attenuates ATO-induced IL- β expression dose-dependently (Figure 5C). Our cytokine beads assay (CBA) data shows an increase in the pro-inflammatory cytokines IL-6, IFN-γ, TNFs, IL-17 A, and IL-10 from the ATO treated cells and BGG (60, 80 μM) treatment found to decrease the expression of pro-inflammatory cytokines in a dose-dependent manner. These data support the potential therapeutic role of BGG to protect against Arsenic induced toxicity (Figure 6).

## Data Availability

Not applicable.

## Sample Availability

Samples of the compounds are not available from the authors upon request.
